# Supplementary figures and images for: Shot-gun proteome and transcriptome mapping of the jujube floral organ and identification of a pollen-specific S-locus F-box gene
Source: PeerJ. 2017 Jul 17;5:e3588. doi: 10.7717/peerj.3588 (PMC5516771; doi:10.7717/peerj.3588)

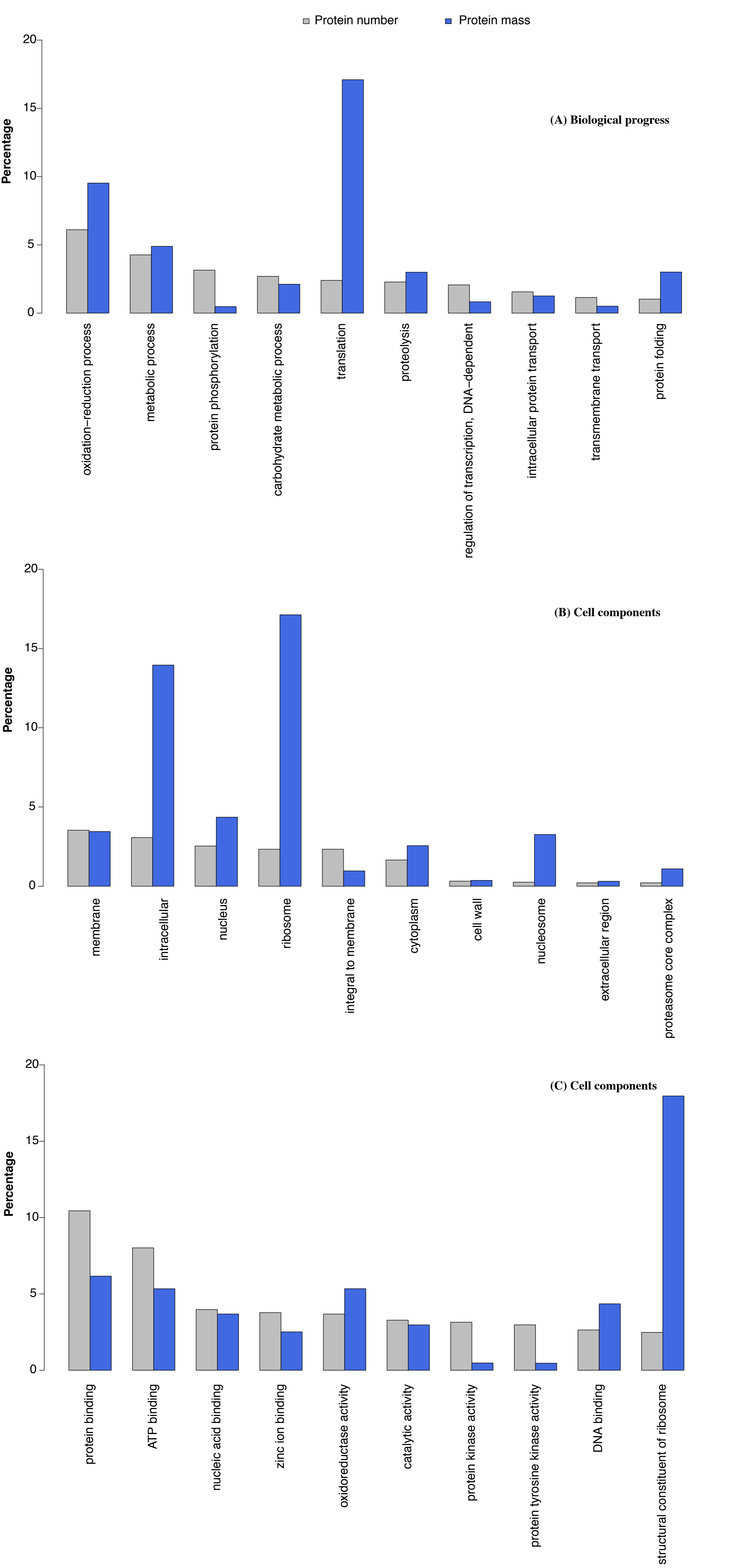

Supplement: Figure S1 [file peerj-05-3588-s001.pdf]
