# Supplementary material for: Shot-gun proteome and transcriptome mapping of the jujube floral organ and identification of a pollen-specific S-locus F-box gene
Source: PeerJ. 2017 Jul 17;5:e3588. doi: 10.7717/peerj.3588 (PMC5516771; doi:10.7717/peerj.3588)
Supplement: Table S1 [file peerj-05-3588-s002.pdf]

Supplementary Table 1 Statistics of clean reads after quality control and the data mapped to genome of 'Junzao'

	**Sample1**	**Sample2***
Total reads	52,842,742	59,415,908
Total mapped reads	47,596,470 (90.07%)	54,791,182 (92.22%)
Multiple mapped reads	1,065,791 (2.02%)	1,157,336 (1.95%)
Uniquely mapped reads	46,530,679 (88.06%)	53,633,846 (90.27%)
Read-1	23,541,145 (44.55%)	26,953,918 (45.36%)
Read-2	22,989,534 (43.51%)	26,679,928 (44.9%)
Reads map to '+' strand	23,247,507 (43.99%)	26,820,210 (45.14%)
Reads map to '-' strand	23,283,172 (44.06%)	26,813,636 (45.13%)
Non-splice reads	34,872,623 (65.99%)	39,232,311 (66.03%)
Splice reads	11,658,056 (22.06%)	14,401,535 (24.24%)

Total reads：total number of filtered clean reads;

Total mapped reads refer to all of those reads mapped to reference genome;

Multiple mapped reads refer to those reads mapped to >1 site,Uniquely mapped reads refer to those reads only mapped to one site;

Splice reads：reads mapped to >1 exons.
